# A conserved 3D pattern in a *Streptococcus pyogenes* M protein immunogen elicits M-type crossreactivity

**DOI:** 10.1016/j.jbc.2023.104980

**Published:** 2023-06-28

**Authors:** Kuei-Chen Wang, Eziz Kuliyev, Victor Nizet, Partho Ghosh

**Affiliations:** 1 Department of Chemistry & Biochemistry, University of California, San Diego, California, USA; 2 Division of Host-Microbe Systems and Therapeutics, Department of Pediatrics, University of California, San Diego, California, USA

**Keywords:** *Streptococcus pyogenes*, M protein, immunogen, C4BP, crossreactivity

## Abstract

Coiled coil–forming M proteins of the widespread and potentially deadly bacterial pathogen *Streptococcus pyogenes* (strep A) are immunodominant targets of opsonizing antibodies. However, antigenic sequence variability of M proteins into >220 M types, as defined by their hypervariable regions (HVRs), is considered to limit M proteins as vaccine immunogens because of type specificity in the antibody response. Surprisingly, a multi-HVR immunogen in clinical vaccine trials was shown to elicit M-type crossreactivity. The basis for this crossreactivity is unknown but may be due in part to antibody recognition of a 3D pattern conserved in many M protein HVRs that confers binding to human complement C4b-binding protein (C4BP). To test this hypothesis, we investigated whether a single M protein immunogen carrying the 3D pattern would elicit crossreactivity against other M types carrying the 3D pattern. We found that a 34-amino acid sequence of *S. pyogenes* M2 protein bearing the 3D pattern retained full C4BP-binding capacity when fused to a coiled coil–stabilizing sequence from the protein GCN4. We show that this immunogen, called M2G, elicited cross-reactive antibodies against a number of M types that carry the 3D pattern but not against those that lack the 3D pattern. We further show that the M2G antiserum–recognized M proteins displayed natively on the strep A surface and promoted the opsonophagocytic killing of strep A strains expressing these M proteins. As C4BP binding is a conserved virulence trait of strep A, we propose that targeting the 3D pattern may prove advantageous in vaccine design.

*Streptococcus pyogenes* (group A *Streptococcus* or strep A) is a globally widespread gram-positive bacterial pathogen that causes a variety of diseases, ranging from mild and self-limiting (*e.g.*, pharyngitis and impetigo) to invasive and deadly (*e.g.*, necrotizing fasciitis and streptococcal toxic shock syndrome) (1). Strep A infection can also lead to autoimmune diseases (*e.g.*, acute rheumatic fever and rheumatic heart disease), which remain serious causes of morbidity and mortality in the developing world (2, 3, 4). Approximately 500,000 deaths occur annually because of diseases caused by strep A (5). At present, there is no vaccine against strep A (6), with one of the major impediments being the sequence variability of its immunodominant surface antigen, the bacterial cell wall–anchored M protein (7, 8, 9, 10, 11).

More than 220 M protein types have been identified (12). The primary sequence of M proteins in general have heptad repeats, which are diagnostic of α-helical coiled coils (13), and structural studies have directly confirmed that M proteins do indeed form parallel and dimeric α-helical coiled coils (14, 15, 16), albeit with functionally significant structural irregularities (17, 18). The sequence of the N-terminal 50 amino acids of the mature form of M proteins (with their signal sequences removed) is hypervariable and defines each of the >220 M types. These N-terminal hypervariable regions (HVRs) elicit protective and opsonizing antibodies (7, 8, 9, 10, 11), whereas other portions of M proteins are often not immunogenic or do not elicit opsonizing antibodies (11, 19). In addition, M protein HVRs do not elicit autoimmune antibodies, whereas other portions of M proteins do (20, 21). While M protein HVRs have highly favorable features as vaccine immunogens, antibody reactivity tends to be type specific and therefore limited to a single M-type strain (22, 23, 24, 25).

Surprisingly, a strep A vaccine immunogen composed of multiple M protein HVRs elicits an M-type crossreactive response. This vaccine immunogen, StreptAnova, consists of 30 different M protein HVRs fused into four separate polyproteins (∼45–50 kDa per polyprotein), and upon immunization of rabbits, it elicits reactivity against these 30 M types as well as crossreactivity against ∼50 M types not included in the immunogen (21, 26, 27). This reactivity promotes opsonophagocytic killing (OPK) of strep A (26, 27). The human immune system also appears to be capable of generating an M-type crossreactive response (28, 29). The basis for M-type crossreactivity of StreptAnova is not known. However, our own work in understanding how human C4b-binding protein (C4BP) binds multiple M protein HVRs provides a plausible explanation (14, 30).

C4BP limits the generation of the major opsonin C3b and thereby functions as a downregulator of the complement system (classical and lectin pathways). Recruitment of C4BP by M protein to the strep A surface is an essential virulence trait, preventing opsonization (OPS), phagocytic uptake, and consequent killing (14, 31, 32, 33, 34, 35, 36, 37, 38). A large-scale study found that 90 of 100 strep A strains of differing M types bound C4BP (38). Because C4BP binding has been attributed overwhelmingly to M protein HVRs (32, 38), these results suggested that C4BP is crossreactive for M protein HVRs. To understand the basis of M-type crossreactivity of C4BP, we determined X-ray crystal structures of four M protein HVRs (M2, M22, M28, and M49), each bound to C4BPα1–2, a fragment of C4BP that is necessary and sufficient to bind M protein HVRs (14). These structures revealed that these M protein HVRs display a similar spatial or 3D pattern of amino acids that contact a common site in C4BP (Fig. S1). The amino acids of this shared 3D pattern are surrounded in space and in primary sequence by a larger number of variable amino acids, and so in effect, the 3D pattern is diluted within the variability of the HVR (30). However, once the 3D pattern was identified, it was recognizable in the primary sequence of M proteins of about 40 of the ∼90 strep A strains (14) that bind C4BP (38).

Based on this observation, we hypothesized that the typical antibody response was M type specific simply because variable amino acids outnumber those in the conserved 3D pattern. However, if an antibody were to bind amino acids of the conserved 3D pattern in one M type, it should then also recognize other M types that have this 3D pattern. Therefore, such an antibody would be M type crossreactive. Notably, 15 of the 30 M protein HVRs in StreptAnova have the C4BP-binding 3D pattern, and correspondingly, 20 of the ∼50 crossreactive M protein types elicited by StreptAnova have the 3D pattern (26, 27), suggesting that at least some of the crossreactivity of StreptAnova is due to recognition of the 3D pattern. Likewise, M-type cross-reactivity observed for three other multi-HVR immunogens may be explained by recognition of the 3D pattern (39, 40, 41). The composition of these three immunogens, which are pentavalent or hexavalent and mostly contain HVRs that are also in StreptAnova, is based on physicochemical properties rather than M-type prevalence in North America and Europe as it is for StreptAnova (26). Together, these results suggest that the C4BP-binding 3D pattern is capable of eliciting M-type crossreactive antibodies.

To test this hypothesis directly, we pursued a detailed proof-of-principle study. We used a short sequence from a single C4BP-binding M protein for immunization. M2 protein was chosen since its binding to C4BP has been studied in detail through mutagenesis (14). A 34-amino acid portion of M2 centered on the 3D pattern maintained full C4BP-binding affinity when fused to the canonical coiled-coil forming protein GCN4 (42). The antiserum evoked by the resulting immunogen, called M2G, was reactive against M2 and crossreactive against a number of C4BP-binding M types but not against M types that do not bind C4BP. The M2G antiserum was not crossreactive against C4b or self-antigens and was competed by C4BP. Reactivity and crossreactivity of the M2G antiserum extended to M proteins displayed natively on the Strep A surface and resulted in the OPK of strep A strains.

## Results

### Minimized C4BP-binding regions of M2 protein

**Figure 1:**
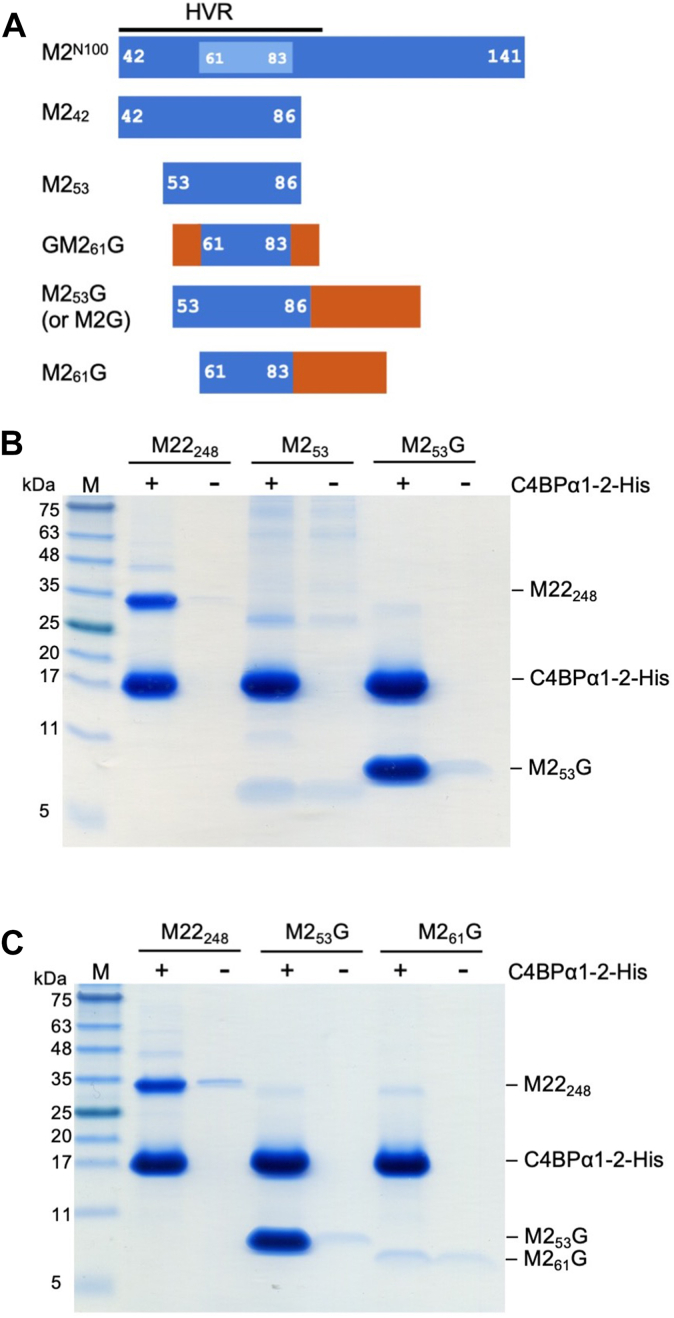
**Binding of minimized M2 protein to C4BPα1–2.***A*, schematic of M2 constructs. M2 in *blue* (with C4BP-contacting amino acids in *cyan*) and GCN4 in *rust*. *B* and *C*, interaction of M2 protein constructs (*B*, M2_53_ and M2_53_G; *C*, M2_53_G and M2_61_G; M22_248_ was used as a positive control) with C4BPα1–2-His at 37 °C, as assessed by a Ni^2+^–NTA agarose coprecipitation assay and visualized by nonreducing Coomassie-stained SDS-PAGE. Bound fractions are shown. Input samples are shown in Fig. S2. Each gel is representative of at least three independent replicates. C4BP, C4b-binding protein.

Our previous structural studies used M2^N100^ (Figs. 1*A* and S1), a protein fragment consisting of the N-terminal 100 amino acids of the mature form of the protein, for cocrystallization with C4BPα1–2 (14). The structure revealed that the C4BP-binding region of M2 protein localized to a span of only 23 amino acids (aa 61–83) within the HVR (Figs. 1*A* and S1). To limit immunoreactivity to the C4BP-binding amino acids of the M2 HVR, we first asked whether a short fragment of M2 protein constituting just the C4BP-binding amino acids would maintain C4BP binding. However, expression of M2 (aa 61–83) by recombinant means in *Escherichia coli* was poor and yielded insufficient quantities of protein for further experiments. We tried longer M2 fragments, either aa 42 to 86 (M2_42_) or 53 to 86 (M2_53_) (Fig. 1*A*). Amino acid 42 is the very N terminus of mature M2 protein, and 53 and 86 are the first and last amino acids, respectively, that are ordered in the crystal structure of M2 bound to C4BPα1–2 (14). Both M2_42_ and M2_53_ were expressed recombinantly in sufficient quantities for further studies. However, neither M2_42_ nor M2_53_ bound His-tagged C4BPα1–2 above background levels (*i.e.*, in the absence of His-tagged C4BPα1–2, Figs. 1*B* and S2, *A* and *B*). A fragment of M22 protein (M22_248_, aa 42–248) was used as a positive control for C4BP binding in this and other experiments, as M22_248_ but not M2^N100^ was distinguishable from C4BPα1–2 on SDS-PAGE. It seemed possible that M2_42_ and M2_53_ were too short to form a dimeric α-helical coiled coil efficiently, a necessity for M protein to bind C4BP (14, 43). To overcome this problem, we fused short sequences from the ideal coiled-coil forming protein GCN4 (42) to M2 protein fragments, maintaining a continuous heptad register between the two (Table S1). We first tried sandwiching M2 (aa 61–83) between single GCN4 heptads (Fig. 1*A*, GM2_61_G) but observed no binding to C4BP (Fig. S2*C*). Next, we tried longer GCN4 coiled-coil sequences of about three or four heptads (23 or 27 aa) fused to the C terminus of M2 aa 53 to 86 or 61 to 83; these fusion constructs were called M2_53_G and M2_61_G, respectively (Table S1). While M2_61_G bound C4BPα1–2 slightly above background level, M2_53_G bound C4BPα1–2 well, with an affinity apparently higher than that of M22_248_ (Fig. 1, *B* and *C*; Fig. S3, *A* and *B*). For simplicity, we refer to M2_53_G as M2G hereafter.

**Figure 2:**
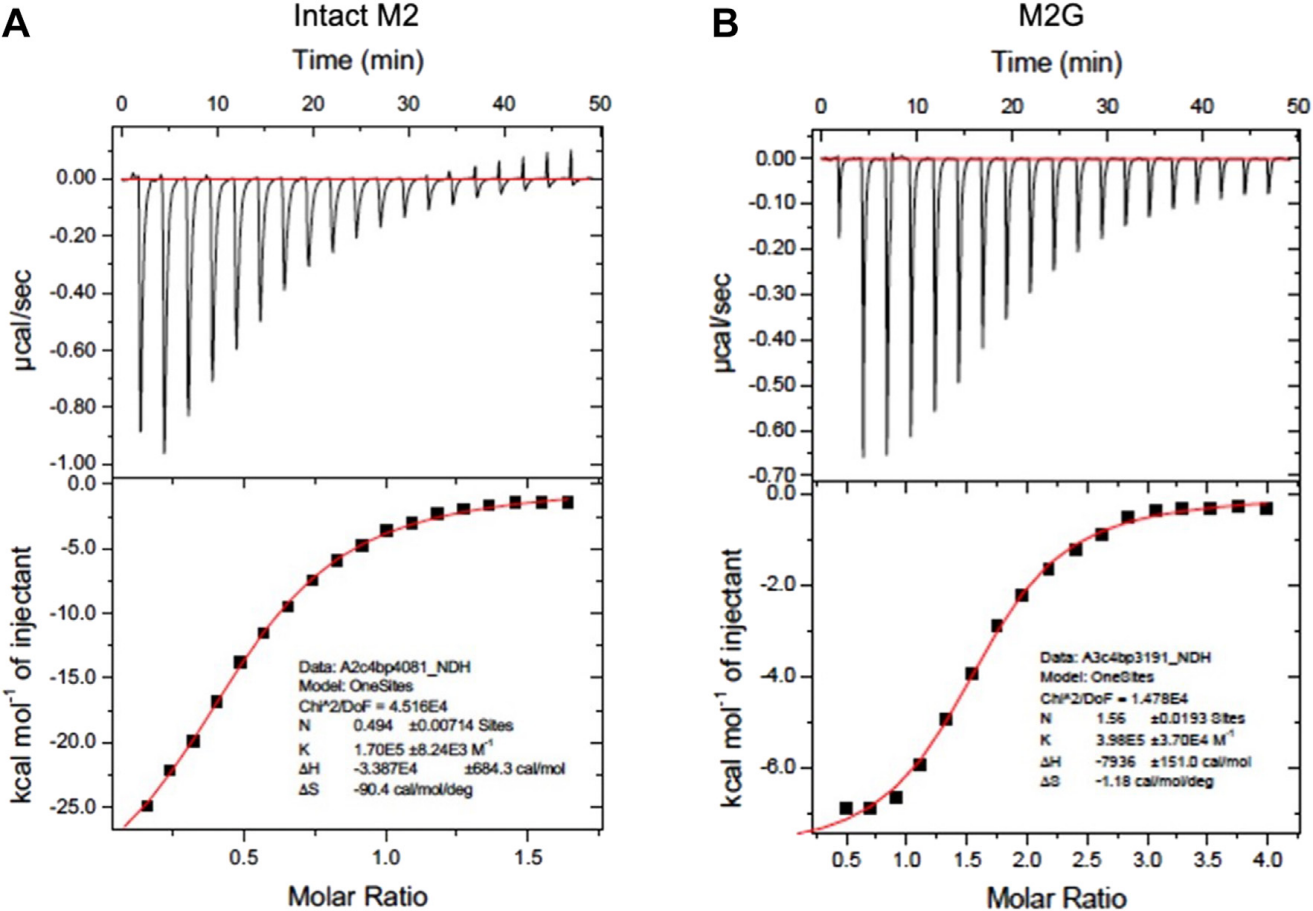
**ITC isotherms and isograms.***A*, intact M2 protein and (*B*), M2G were titrated into a solution of C4BPα1–2. The *top half* of each panel shows isotherms and the *bottom half* isograms. The binding curves were fit using a single-site model with the Origin software package. Each panel is representative of three experimental replicates. C4BP, C4b-binding protein; ITC, isothermal titration calorimetry.

We then asked whether M2G recapitulated the C4BP-binding affinity of intact M2 protein. Isothermal titration calorimetry (ITC) was carried out and showed that the *K*_*D*_ of C4BPα1–2 bound to intact M2 protein was identical to that of C4BPα1–2 bound to M2G, 4 μM (Fig. 2 and Table 1). Thus, M2G, which contains only 34 aa of M2, possessed the full C4BP-binding affinity of intact M2 protein. Furthermore, these results suggested that GCN4 aided the coiled-coil dimerization of this M2 region to restore its C4BP-binding ability.

**Table 1:** ITC analysis of M protein–C4BPα1–2 interaction

Protein	*K*_*D*_ (μM)	N^a^	Stoichiometry (C4BP:M)	ΔH (kcal/mol)	–TΔS (kcal/mol)
Intact M2	4 ± 2	0.50 ± 0.01	2:1^b^	−24 ± 10	17 ± 10
M2G	4 ± 1	0.51 ± 0.01	2:1	−27 ± 6	20 ± 6

a N, binding stoichiometry of the M protein.

b Two molecules of C4BPα1–2 per 1 M protein dimer.

### M2G as an immunogen

Having identified that M2G recapitulated C4BP binding, we asked whether it was sufficient to evoke an immune response that was crossreactive against M protein types that carry the 3D pattern. Rabbit polyclonal antibodies were raised against M2G and assayed for reactivity against various recombinant M proteins. While M2, M22, M28, and M49 HVRs present similar 3D patterns of amino acids that are complementary to C4BP, these spatial patterns are exhibited differently in the heptad repeats of their primary sequences (14) (Fig. S1). The heptad patterns of M2 and M49 HVRs are similar to one another and belong to one subset, the M2/M49 sequence pattern; and the M22 and M28 HVR patterns are similar to one another and belong to a second subset, the M22/M28 sequence pattern (14). We chose M protein types from each pattern that are prevalent in human infectious disease epidemiology (44, 45). For the M2/M49 group, these were M2, M49, M73, M77, and M89 proteins, and for the M22/M28 group, these were M4, M11, M22, M28, M44, and M81 proteins (Table S2). As negative controls, we used M1, M5, and M6 proteins, which do not bind C4BP and lack the 3D pattern.

We expressed and purified constructs constituting the N-terminal 100 amino acids of the mature forms of these M proteins. Binding to C4BP had not been directly evaluated for some of the M proteins. For these, an ELISA for C4BP binding was carried out (Fig. S4). All M^N100^ constructs (*i.e.*, N-terminal 100 aa of the mature form) belonging to the M2/M49 or M22/M28 pattern bound C4BP at a statistically significant level, except for M77^N100^, which bound C4BP at the level of the negative control M5^N100^ construct.

**Figure 3:**
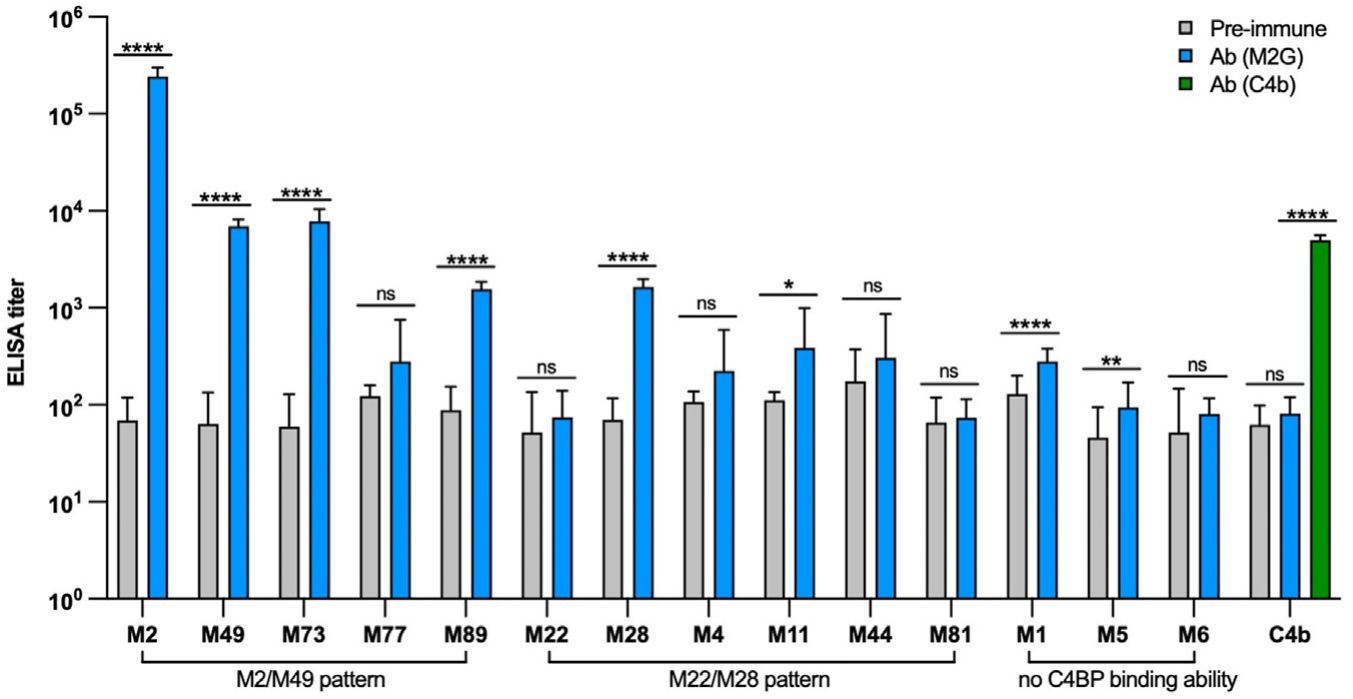
**Reactivity and crossreactivity of M2G antiserum.** Titers of preimmune and immune M2G sera against M^N100^ constructs (N-terminal 100 amino acids of the mature form of the protein) or C4b, as determined by ELISA. M^N100^ constructs or C4b were adhered to ELISA plate wells, and varying dilutions of preimmune serum (*gray*), M2G serum (*blue*), or anti-C4b (*green*) antibodies were added. Bound antibodies were detected at absorbance at 450 nm with HRP-conjugated secondary antibodies. The titer is defined as the serum or antibody dilution that yielded half-maximal absorbance at 450 nm, based on sigmoidal curve fits of the dilution data, which are shown in Fig. S5. All measurements were in triplicate, and experiments were performed at least two independent times. Statistical analyses were performed by Student’s *t* tests and one-way ANOVA for M^N100^ constructs and C4b, respectively; ∗*p* < 0.05, ∗∗*p* < 0.01, ∗∗∗*p* < 0.001, ∗∗∗∗*p* < 0.0001, *p* > 0.05 (not significant, ns). HRP, horseradish peroxidase.

We next tested the reactivity and crossreactivity of the M2G antiserum. As expected, the M2G antiserum recognized M2^N100^ well, with an antibody titer that was significantly greater than that of preimmune serum (>10^5^ *versus* <10^2^) (Figs. 3 and S5). The M2G antiserum was crossreactive with titers >10^3^ against all the M^N100^ constructs belonging to the M2/M49 pattern (*i.e.*, M49, M73, and M89), except for M77^N100^, which as noted previously did not bind C4BP. For the M22/M28 pattern, the M2G antiserum was crossreactive with a titer >10^3^ against only M28^N100^. Statistically significant crossreactivity was also seen for M11^N100^, but the titer was low (<10^3^). For the remaining members of M22/M28 group (M22^N100^, M4^N100^, M44^N100^, and M81^N100^), the titer of the M2G antiserum was low (<10^3^) and not significantly different from that of the preimmune serum (Figs. 3 and S5). The titer of the M2G antiserum was uniformly low against M^N100^ constructs of M proteins that are known not to bind C4BP (*i.e.*, <10^3^ for M1 and <10^2^ for M5 and M6) (Figs. 3 and S5). These results indicate that a single M protein antigen can elicit crossreactivity based on the C4BP-binding 3D pattern.

Because both M protein HVRs and C4b are bound by nearly the same site on C4BP (34), we asked whether the M2G antiserum possessed unwanted crossreactivity against C4b. The M2G antiserum did not crossreact against C4b, as evaluated by ELISA with C4b adhered to a solid substrate (Fig. 3, titer <10^2^). The conformational integrity of C4b adhered to the solid substrate was verified by its recognition by an anti-C4b antibody (Figs. 3 and S5*C*). These results are consistent with the observation that M protein HVRs and C4b differ in their binding mode for C4BP (34).

While autoreactivity is not attributed to M protein HVRs, this issue remains a general concern for vaccines based on M proteins (46). To evaluate the reactivity of the M2G antiserum against human tissues affected in strep A autoimmune sequelae, Western blot analysis was performed with normal adult human brain (HB) tissue lysate and human heart (HH) tissue lysate. Because autoreactivity can be due to portions of M proteins outside the HVR, we also raised rabbit antibodies against intact M2 protein and compared the crossreactivity of the M2G antiserum against that of the intact M2 protein antiserum. The M2G antiserum reacted against intact M2 protein but not HB or HH (Fig. S6*A*). In contrast, the antiserum raised against intact M2 protein reacted against intact M2 and both HB and HH (Fig. S6*B*). These results suggest that the M2G immunogen does not elicit crossreactivity against human tissues, whereas intact M2 protein has the potential to do so. These results are also consistent with M protein HVRs not eliciting autoreactive antibodies (21).

We asked whether the pattern of M protein crossreactivity described previously was limited to a single rabbit or reproducible in a second rabbit. To this end, a second rabbit was immunized with M2G, and the crossreactivity of this second rabbit’s M2G antiserum was examined (Figs. S7 and S8). The reactivity and cross-reactivity patterns were almost identical with a Pearson correlation coefficient of 0.998. Some small differences were evident. For example, for the M2/M49 pattern, the relative crossreactivity to M89^N100^ was lower in the second rabbit than in the first, and in the M22/M28 pattern, low-titer crossreactivity to M44^N100^ but not M11^N100^ was statistically significant. Experiments were continued using the antiserum from the first rabbit.

As the M2G antigen contains sequences from both M2 protein and GCN4, we asked whether crossreactivity was due to antibodies specific for the M2 portion as opposed to the GCN4. We focused on M protein constructs that yielded the highest titers (>10^3^), namely M2^N100^, M49^N100^, M73^N100^, M89 ^N100^, and M28^N100^ (Fig. 3). Reactivity of the M2G antiserum to these M protein constructs was competed with increasing concentrations of either M2G or M6G (Table S1). The latter consists of a portion of M6 protein (aa 56–89) fused to nearly the identical portion of GNC4 in M2G (Table S1). For all M^N100^ constructs except for M49^N100^, competition occurred with M2G but not M6G, even when M6G was used at greater than twofold higher concentration (Fig. S9*A*). The integrity of M6G as a competitor was demonstrated by adhering His_6_-tagged M6G to ELISA plates and using soluble His_6_-tagged M6G as a competitor (Fig. S9*B*). These results indicated that for M2^N100^, M73^N100^, M28^N100^, and M89^N100^, crossreactive antibodies were specific to the M2 portion of the M2G immunogen. For M49^N100^, both M2G and M6G competed against the M2G antiserum for binding (Fig. S9*A*), suggesting that some or all crossreactive antibodies against M49^N100^ were specific to the GCN4 portion of the M2G. M49 protein was eliminated from further studies.

We tested the hypothesis that the M2G antiserum was directed against the C4BP-binding pattern by asking whether C4BP would compete with the M2G antiserum. Incubation of intact C4BP with M2^N100^, M73^N100^, M28^N100^, and M89^N100^ significantly decreased their interaction with the M2G antiserum, whereas incubation with fibrinogen, as a negative control, had no effect (Fig. S10). Fibrinogen does not bind to these M protein types (47). Competition by C4BP was partial, suggesting that the M2G antiserum had greater affinity for M protein constructs than C4BP, consistent with the micromolar affinity of C4BP for M2 protein reported previously. While intact C4BP with its seven potential M protein–binding sites was used in this experiment, both C4BP and M protein constructs were soluble, and thus, no picomolar avidity interaction was possible (47). These results are consistent with the conserved 3D pattern eliciting crossreactivity.

### Strep A surface binding and opsonophagocytic activity of M2G antiserum

**Figure 4:**
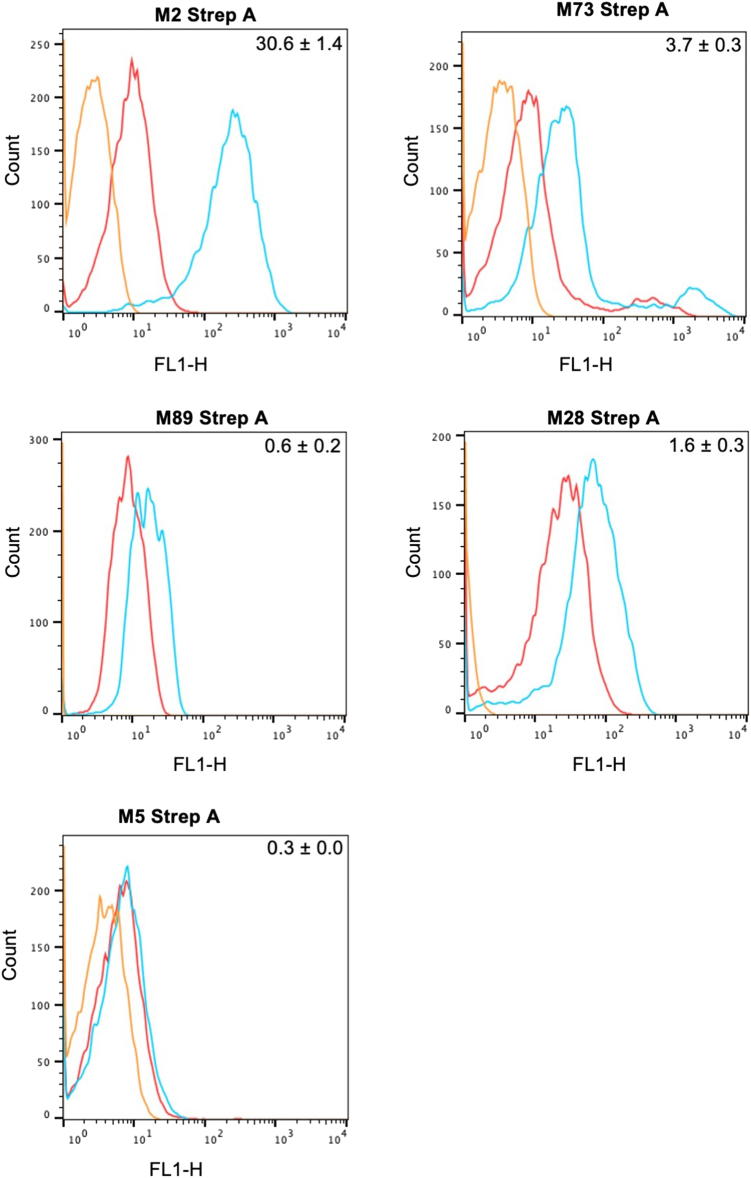
**M2G antiserum binding to strep A.** Binding of preimmune serum and M2G antiserum to strep A M2, M73, M89, M28, and M5 strains, assessed by flow cytometry. Histograms show fluorescent intensities from preimmune serum (*red*), M2G antiserum (*blue*), and anti-rabbit immunoglobulin G (IgG) antibody control (*orange*, 2° antibody only). Each histogram is representative of three independent replicates. Numbers on the *top right corner* of each panel is the difference between the geometric mean of the fluorescent signal of the M2G antiserum and that of preimmune serum, normalized by that of the preimmune serum. The geometric mean of the fluorescent signal from the 2° antibody was first subtracted from these values. The fluorescent signal of preimmune serum (pre) and M2G antiserum (Ab(M2G)), after subtraction of background fluorescence from the 2° antibody, is listed in Table S3.

To assess whether the M2G antiserum recognized M proteins on the bacterial surface, we carried out flow cytometry on whole, living strep A strains of differing M types. In line with results using purified proteins, the M2G antiserum bound the surface of an M2 strain to a significantly higher extent than did the preimmune serum (Fig. 4 and Table S3). Likewise, the M2G antiserum displayed significant crossreactivity compared with the preimmune serum against strep A M73, M89, and M28 strains. No crossreactivity was seen against an M5 strain, which does not bind C4BP. Overall, these results confirm that the M2G antiserum recognizes M proteins in their native conformation on the strep A surface.

**Figure 5:**
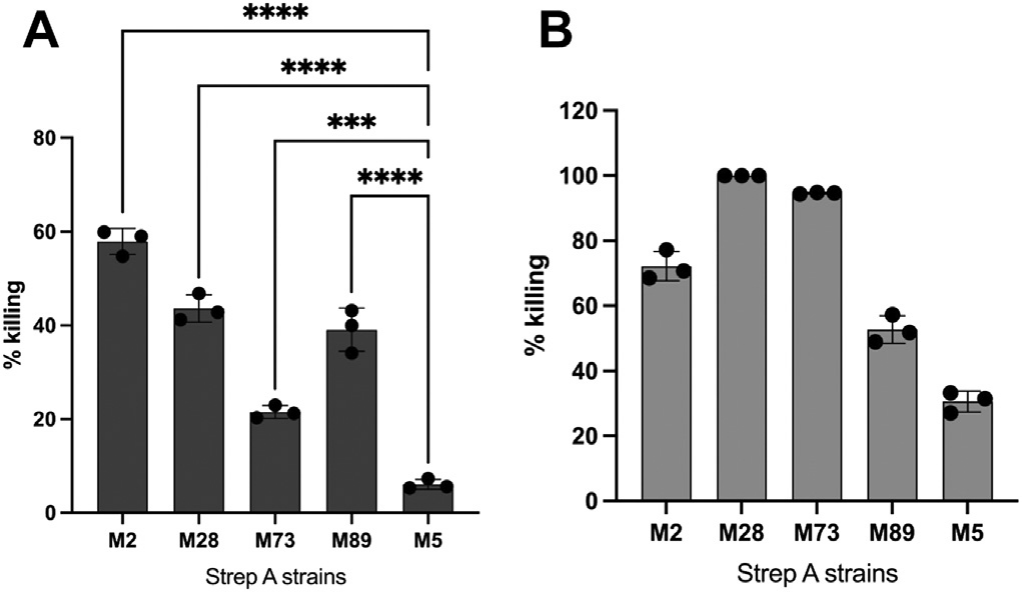
**Bactericidal activity of M2G antiserum.** Opsonophagocytic killing promoted by M2G antiserum (*A*) or IVIG (*B*) of strep A M2, M28, M73, M89, and M5 strains. The assay was performed with DMF-differentiated HL-60 cells and baby rabbit complement. All experiments were carried out in triplicate and performed three independent times. Statistical analysis for the M2G antiserum was performed by one-way ANOVA; ∗*p* < 0.05, ∗∗*p* < 0.01, ∗∗∗*p* < 0.001, ∗∗∗∗*p* < 0.0001, *p* > 0.05 (not significant, ns). DMF, dimethylformamide; IVIG, intravenous immune globulin.

Antibodies against M protein HVRs elicit opsonophagocytic antibodies (7, 8, 9, 10, 11). To verify that this is the case for the M2G antiserum, we evaluated whether the M2G antiserum promoted OPK of reactive and crossreactive strep A strains. For the OPK assay, we used cultured HL-60 cells differentiated to have a neutrophil-like phenotype, along with baby rabbit serum as the source of complement (48). While neutrophil-like HL-60 cells are not nearly as potent killers as primary neutrophils (49), they offer advantages over primary neutrophils or whole human blood as in the classical Lancefield assay (24). Most importantly, individual variation in complement and neutrophil activity is eliminated in the HL-60 assay, as is the existence of immunity against various M types (50). The HL-60 assay has been proposed as a standard means for evaluating strep A vaccine candidates (48, 50). We focused on the M types against which the greatest reactivity and crossreactivity had been demonstrated—M2, M73, M89, and M28—and used an M5 strain as a negative control. As validation of the assay, we used intravenous immune globulin (IVIG), a concentrated pool of human antibodies, which resulted in killing of all five strains (Fig. 5). We found that the M2G antiserum had substantial OPK activity against M-type strains that it reacted with or was crossreactive against. The highest OPK activity was for the M2 strain (57.9 ± 2.8%) followed by the M28 (43.6 ± 2.9%), M89 (39.1 ± 4.5%), and M73 strains (21.5 ± 1.4%) (Fig. 5). These values were statistically significant compared with the OPK activity against the M5 strain (6.1 ± 1.1%). These results confirmed that antibodies directed against M protein HVRs promote OPK.

## Discussion

We set out to test the hypothesis that directing the antibody response to the C4BP-binding 3D pattern in one M protein type would lead to crossreactivity against other M protein types that share the 3D pattern. The design was to maximize the contribution of the 3D pattern to the immunogen and correspondingly minimize that of variable amino acids. The well-studied M2 protein was chosen for these studies (14). Short segments of M2 protein containing the 3D pattern (*e.g.*, aa 53–86) bound C4BPα1–2 poorly, likely because they formed unstable coiled coils. This finding may reflect the presence of charged amino acids at some of the core *a* positions of the coiled-coil heptad (14). Stabilization of coiled-coil structure through fusion of these M2 segments to portions of GCN4 (*i.e.*, in M2G) restored C4BP binding. While the 3D pattern in M2 protein appeared to start at aa 61 (14), inclusion of amino acids upstream of this position had a significant effect on C4BP binding, leading to a *K*_*D*_ that matched that of intact M2 protein. In the crystal structure, these upstream amino acids contact a crystallographically related C4BPα1–2 molecule (14). Based on these results, it is likely that the contacts made by these upstream amino acids to C4BP are not a crystallization artifact but instead a *bona fide* interaction.

The M2G immunogen elicited a crossreactive response against M73 and M89 proteins from the M2/M49 pattern. Crossreactivity was also observed against M49 protein, but this was in part or entirely attributable to antibodies that were specific to GCN4 rather than M2 portions in the M2G immunogen. No notable sequence similarity exists between M49 and GCN4, and thus, the basis for this surprising result requires further investigation. No crossreactivity was seen to M77 protein, which despite having an unambiguous M2/M49 sequence pattern (14) (Fig. S1), did not bind C4BP. In the case of some M proteins (51), C4BP binding is significantly enhanced by the concurrent binding of Fc domains from human immunoglobulin G (IgG). M77 protein may require this additional interaction to bind C4BP. Alternatively, C4BP binding may be conferred by a portion of M77 protein outside the HVR or another bacterial surface–associated protein (32). The M2G immunogen elicited a strong crossreactive against only one member of the M22/M28 pattern, M28 protein. While the spatial arrangement of C4BP-interacting amino acids (*i.e.*, 3D pattern) is similar for both M2/M49 and M22/28 sequence patterns (14), the different positioning of M protein α-helices between these two patterns appears to influence crossreactivity (Fig. S1). Clearly, further work is required to increase the scope of crossreactivity of a 3D pattern–based immunogen. It may be worthwhile in the future to explore the use of consensus C4BP-binding sequences from the M2/M49 and M22/M28 patterns rather than the sequence of a single M type and a means for stabilizing the coiled coil without fusion to GCN4 or other proteins.

The M2G antiserum crossreacted against M proteins in their native conformation on the strep A surface, which was likely favored by an immunogen that retained C4BP binding and hence native conformation. Furthermore, the M2G antiserum promoted the OPK of strep A strains of multiple M types, consistent with results showing that M protein HVRs evoke opsonic antibodies (7, 8, 9, 10, 11). The range of killing of strep A by HL-60 cells because of crossreactivity seen here (22–44%) was similar or slightly better than that observed in another study (16–41%), in which peptides derived from multiple M protein HVRs served as the immunogen (52). Notably, the crossreactivity we observed correlated better with C4BP binding than sequence identity (Table S2). For example, M77 protein, which did not bind C4BP and was not recognized by the M2G antiserum, has 59% identity with the M2G immunogen, but M28 protein, which did bind C4BP and was recognized, has only 35% identity. OPK of the M28 strep A strain at 44% was the highest of all the crossreactive interactions observed.

These results provide evidence that the conserved C4BP-binding 3D pattern elicits antibodies that crossreact against M protein types that have the 3D pattern and promote the OPK of such strep A strains. Significantly, the recruitment of C4BP to the strep A surface is an essential virulence trait for numerous strep A strains (33, 35, 37), and thus, escape from a broadly protective antibody that targets the C4BP-binding 3D pattern through further sequence variation may be limited by pressure to maintain C4BP interaction during infection (30). In effect, the C4BP-binding 3D pattern is an Achilles’ heel of many M protein types. These results provide impetus to pursue further experiments aimed at optimizing an immunogen based on the C4BP-binding 3D pattern.

## Experimental procedures

### S. pyogenes

The following *S. pyogenes* clinical isolates from the US Centers for Disease Control and Prevention were used in this study: *emm2* (strain 3752-05), *emm5* (strain 3292-05), *emm28* (strain 4039-05), *emm49* (strain 3487-05), *emm73* (strain 3962-05), and *emm89* (strain 4264-05). *S. pyogenes* was grown statically in Todd-Hewitt broth (THB; BD) supplemented with 1% yeast extract (Gibco) overnight at 37 °C, and afterward subcultured in the same medium until midlogarithmic growth phase (absorbance at 600 nm = 0.4–0.6).

### Cloning and DNA manipulation

Coding DNA sequences for intact mature M1, M2, M4, M5, M22, M28, and M49 proteins were cloned, as described previously (14, 16), from *S. pyogenes* strains M1 (strain 5448), M2 (AP2), M4 (Arp4), M5 (Manfredo), M22 (Sir22), M28 (strain 4039-05), and M49 (NZ131), respectively, and ligated into pET-28b vector (Novagen) or a modified pET-28a vector (Novagen) that had encoded an N-terminal His_6_-tag followed by a PreScission protease (GE Healthcare) cleavage site. Truncated forms of these proteins were subcloned from these vectors. The coding DNA sequence for GCN4 was subcloned from *Saccharomyces cerevisiae*. The coding sequence of the N-terminal 100 amino acids of M6, M73, M77, M89, M11, M44, and M81 proteins were chemically synthesized (Integrated DNA Technologies, Inc) and inserted into the modified pET-28a vector. M protein–GCN4 fusion constructs were produced by strand overlap extension PCR and ligated into the modified pET-28a vector. Protein sequences of M protein–GCN4 fusion constructs are listed in Table S1.

### Protein expression and purification

M proteins were expressed in *E. coli* BL21 (Gold) and purified as previously described (14, 16), except that imidazole was not included in the lysis buffer. C4BPα1–2 was expressed in *E. coli* Rosetta 2 (Novagen). The protein was purified and refolded as previously described (53) with minor modifications. Specifically, bacteria were lysed with a C-5 Emulsiflex (Avestin). After refolding and dialysis, C4BPα1–2 was applied to a HiTrap Q HP column (GE Healthcare) and eluted using a 0 to 1 M NaCl gradient in 50 mM Tris, pH 8.5.

### Coprecipitation assays

His_6_-tagged C4BPα1–2 (150 μg) was mixed with M protein constructs (molar ratio 1:1.2) in 50 μl PBS at 37 °C for 30 min under rotation. Ni^2+^–NTA agarose beads (100 μl of 50% slurry), pre-equilibrated with PBS, were then added to the protein mix and incubated at 37 °C for 40 min under rotation. The beads were washed three times with 0.5 ml of PBS supplemented with 15 mM imidazole and eluted with 40 μl of PBS supplemented with 300 mM imidazole. Proteins in the input and eluted fractions were resolved by nonreducing SDS-PAGE and visualized by Coomassie staining.

### ITC

ITC experiments were performed at 23 °C on a ITC200 microcalorimeter (MicroCal) with PBS as the assay buffer. Titrations were carried out with 300 to 500 μM intact M2 or M2G, which was loaded in the injection syringe (40 μl), and 30 to 50 μM C4BPα1–2, which was loaded in the sample cell (∼250 μl). A typical titration experiment consisted of 19 injections of 2 μl over a duration of 4 s; each injection was separated by 150 s. The cell stirring speed was 1000 rev/min. Raw data were collected, and binding curves were fitted using a single-site model with Origin software (MicroCal).

### Rabbit polyclonal antisera

Rabbit polyclonal antisera were raised commercially (Pocono Rabbit Farm & Laboratory) against 200 μg of purified M protein constructs (M2G or intact M2 protein). An initial immunization in complete Freund’s adjuvant was carried out, followed by three boosts with 100 μg purified protein in incomplete Freund’s adjuvant on days 14 and 28, and 50 μg purified protein in incomplete Freund’s adjuvant on day 56. A large bleed was performed on day 70 to obtain serum.

### ELISA

#### Determination of antibody titers

Purified M^N100^ protein constructs at 1 μg/ml were coated in the wells of 96-well microtiter plates (Corning) in carbonate buffer (50 mM Na_2_CO_3_–NaHCO_3_, pH 9.6) overnight at 4 °C. All subsequent procedures were performed at room temperature (RT). Wells were washed three times in TBST (150 mM NaCl, 50 mM Tris, pH 8.0, and 0.1% Tween-20) and blotted dry after the preceding step and after all steps described later. Wells were blocked with 0.1% bovine serum albumin (BSA) in TBST for 1 h and then incubated with 100 μl of rabbit preimmune or immune serum (serially diluted in 0.1% BSA/TBST) for 1.5 h. In the case of competition experiments, varying concentrations of M2G or M6G were incubated with the immune serum for 10 min prior to addition to wells. One hundred microliter horseradish peroxidase (HRP)–conjugated goat anti-rabbit IgG (H + L) (Southern Biotech) at 1:4000 dilution in 0.1% BSA/TBST was added and incubated for 1 h. For detection, 100 μl TMB substrate (BD Biosciences) was added and incubated for 10 min (protected from light), followed by addition of 50 μl of 2 N sulfuric acid to stop the reaction. Triplicate data from at least two independent experiments were normalized and combined. The normalization factor was the mean absorbance at 450 nm of the first dilution point (ranging from 10^1^ to 10^3^ depending on the M protein) of a particular independent experiment. The combined values were fit to a sigmoidal curve using GraphPad Prism (GraphPad Software, Inc). Antibody titers were defined as the interpolated serum dilution level that yielded 50% of the maximum absorbance at 450 nm. Statistical analysis was performed using the Student’s *t* test to compare immune and preimmune sera.

#### Detection of C4b

An ELISA was carried out as aforementioned, except that C4b (Millipore) at 1 μg/ml was coated in ELISA plate wells, and detected by incubation for 1 h with 100 μl anti-C4b polyclonal antibodies (Thermo Fisher) at 1:4000 dilution in 0.1% BSA/TBST, followed by incubation for 1 h with goat antichicken IgY-HRP (Santa Cruz Biotechnology) at 1:2500 dilution in 0.1% BSA/TBST.

#### His_6_-M6G *versus* His_6_-M6G competition

ELISAs were carried out as aforementioned, except that His_6_-tagged M6G at 1 μg/ml in PBS was coated in the wells of 96-well microtiter plates. Soluble His_6_-tagged M6G at varying concentrations was then incubated for 10 min with HRP-conjugated mouse anti-His antibody (BioLegend) diluted 1:2000 in 0.1% BSA/TBST. Wells were then incubated for 1.5 h with 100 μl of this solution.

#### M protein–C4BP interaction

ELISAs were carried out as aformentioned, except that intact C4BP (Complement Technology) at 10 μg/ml in PBS was coated in the wells of 96-well microtiter plates. Wells were incubated for 1.5 h with 10 μg/ml of His_6_-tagged M proteins (N-terminal 100 amino acids, diluted in 1% BSA/TBST). Wells were then incubated with 100 μl HRP-conjugated mouse anti-His antibody (1:2000 dilution in 1% BSA/TBST; BioLegend).

#### C4BP competition with M2G antiserum

ELISAs were carried out as aforementioned, except that M2G antiserum (10^3^ dilution) in PBS was coated in the wells of 96-well microtiter plates overnight at 4 °C. His_6_-tagged M protein constructs (0.5 μM) were added alone or with 0.7 μM C4BP or 5 μM fibrinogen (EMD Millipore), with which they had first been incubated for 10 min, to wells and incubated for 1.5 h. Wells were then incubated with 100 μl HRP-conjugated mouse anti-His antibody (1:2000 dilution in 1% BSA/TBST).

### Human tissue crossreactivity

Twenty micrograms of normal adult HB tissue lysate (Novus Biologicals) or heart tissue lysate (Novus Biologicals) was resolved on 4 to 20% gradient SDS-PAGE (Bio-Rad) and transferred to a polyvinylidene fluoride membrane (Millipore) for immunoblotting. Membranes were blocked with 5% BSA in TBST at RT for 1 h and then incubated with rabbit antisera (1:1000 dilution in 5% BSA/TBST) at RT for 1 h. Membranes were washed three times by TBST for 5 min each. Membranes were then incubated with HRP-conjugated goat anti-rabbit IgG (H + L) (1:4000 dilution; Southern Biotech) at RT for 1 h, and SuperSignal West Pico Chemiluminescent Substrate (Thermo Fisher Scientific) was then added. The resulting chemiluminescence was recorded on a ChemiDoc XRS+ imaging system (Bio-Rad).

### Antiserum binding to *S. pyogenes*

*S. pyogenes* was grown to midlogarithmic phase, washed in PBS, and blocked with 10% heat-inactivated donkey serum in PBS (Sigma–Aldrich) at RT for 1 h. Heat-inactivated M2G antiserum or preimmune serum was added to *S. pyogenes* to 1% final volume and incubated at RT for 1 h. After washing once in PBS, samples were incubated in 1:200 dilution of donkey anti-rabbit IgG antibody with Alexa Fluor 488 conjugation (BioLegend) at RT for 30 min (protected from light). Samples were then washed once in PBS, resuspended in PBS, and analyzed by flow cytometry (BD FACSCalibur). Fluorescent signal intensity was analyzed using FlowJo software (Tree Star, Inc).

### HL-60 OPK assay

The OPK assay was performed as previously described (48, 50) with some modifications. HL-60 cells (CCL-240; American Type Culture Collection) were cultured in RPMI medium (RPMI1640 with 1% l-glutamine [Corning] and 10% heat-inactivated fetal bovine serum [Gibco]). Differentiation into neutrophil-like cells was carried out through incubation for 4 to 5 days and with a cell density of 4 × 10^5^ cells/ml in RPMI medium supplemented with 0.8% dimethylformamide. The phenotype of differentiated HL-60 cells was assessed by flow cytometry using mouse antihuman CD35 phycoerythrin-conjugated antibody (BioLegend) and mouse antihuman CD71 allophycocyanin-conjugated antibody (BioLegend). Differentiated cells were used in the OPK assay if >55% of cells were CD35^+^ and <15% of the cells were CD71^+^. Prior to use in the assay, differentiated HL-60 cells were washed first in Hank’s balanced salt solution (HBSS) without Ca^2+^/Mg^2+^ (Gibco) and then in HBSS with Ca^2+^/Mg^2+^ (Gibco) and resuspended at a concentration of 1 × 10^7^ cells/ml in fresh OPS buffer (HBSS with Ca^2+^/Mg^2+^, 0.1% gelatin, 5% heat-inactivated pig serum [Sigma–Aldrich], 1 mg/ml human fibrinogen [Millipore], and 10 U/ml heparin [Sigma–Aldrich]).

Prior to carrying out the OPK assay, *S. pyogenes* strains were passaged through HL-60 cells, as follows. *S. pyogenes* was grown to midlogarithmic phase and then diluted in THB to 3000 to 10,000 colony-forming unit (CFU)/ml. About 10 μl of *S. pyogenes* were incubated with 50 μl heat-inactivated normal rabbit serum (NRS; Pocono Rabbit Farm & Laboratory) per well in a round-bottom 96-well plate (Corning) at RT for 30 min, followed by the addition of 40 μl of active baby rabbit complement (BRC; Pel-Freez) and 100 μl of differentiated HL-60 cells to each well. The plate was then sealed with aluminum film (AlumaSeal II AF100; Excel Scientific) and incubated at 37 °C for 2 h with end-over-end rotation. The final concentration of BRC in the reaction mixture was 5 to 20% in OPS buffer, with the specific value dependent on the *S. pyogenes* strain (such that nonspecific killing, as described later, was <35%). After 2 h incubation, the plate was placed on ice for 30 min to stop the activity of HL-60 cells. After mixing thoroughly, 10 μl from each well was spotted on THB agar plates, which were tilted immediately to spread the bacteria in drips across the plates. The plates were incubated overnight at 37 °C, and *S. pyogenes* colonies were recovered. This procedure was carried out a second time to yield a total of two passages for each strain.

The OPK assay was carried out as aforementioned with twice-passaged *S. pyogenes*. Bacteria were incubated with HL-60 cells, heat-inactivated NRS, and heat-inactivated BRC (control A); HL-60 cells, heat-inactivated NRS, and active BRC (control B); or HL-60 cells, 50 μl heat-inactivated M2G antiserum or 50 μl heat-inactivated IVIG 10%; Octapharma USA, Inc), and active BRC. After overnight incubation of THB agar plates, CFUs were enumerated.

The percentage of killing was calculated as ([CFU of control B – CFU of M2G antiserum or IVIG]/CFU of control B) × 100. The percentage of nonspecific killing was calculated as ([CFU of control A – CFU of control B]/CFU of control A) × 100. The number of bacterial generations was calculated by comparing the total CFU of control B to the CFU in the inoculum. Only assays in which the level of nonspecific killing was <35%, the number of bacterial generations in control B was >4, and the CFUs of controls A and B between 50 and 200 were considered. Statistical analysis was performed using one-way ANOVA to compare M2, M28, M73, and M89 strains to M5.

## Data availability

All data are included in the article.

## Supporting information

This article contains supporting information.

## Conflict of interest

The authors declare that they have no conflicts of interest with the contents of this article.
