# Supplementary material for: A conserved 3D pattern in a Streptococcus pyogenes M protein immunogen elicits M-type crossreactivity
Source: J Biol Chem. 2023 Jun 28;299(8):104980. doi: 10.1016/j.jbc.2023.104980 (PMC10400905; doi:10.1016/j.jbc.2023.104980)
Supplement: Supporting Figures S1–S10 and Tables S1–S3 [file mmc1.pdf]

A conserved 3D pattern in a *Streptococcus pyogenes* M protein immunogen elicits M type cross-reactivity

Kuei-Chen Wang[1,a], Eziz Kuliyev[1.a], Victor Nizet[2], and Partho Ghosh[1,*]

[1]Department of Chemistry & Biochemistry, University of California, San Diego, La Jolla, CA 92093,

[2]Division of Host-Microbe Systems and Therapeutics, Department of Pediatrics, University of California, San Diego, La Jolla, CA 92093

*To Whom Correspondence Should be Addressed: pghosh@ucsd.edu

[a]These authors contributed equally.

Supplementary Figure Legends

Supplementary Figures S1-S10

Supplementary Tables S1-S3

Supplementary Figure Legends

Figure S1. C4BP-binding 3D Pattern in M protein HVRs.

a. Sequence alignment of M protein HVRs belonging to M2/M49 and M22/M28 patterns. Only M proteins investigated here are shown. M2, M49, M22, and M28 are bolded as their structures in complex with C4BPα1-2 are known. Red amino acids denote contacts to C4BPα1-2 from one of the α-helices of the M protein coiled coil; blue amino acids denote contacts to C4BPα1-2 from the other α-helix of the M protein coiled coil. Numbers preceding the sequence correspond to amino acid position. Heptad positions are indicated at the top, and *a* and *d* core heptad positions are shaded.

b. Structure of M2 and M28 HVRs from their complexes with C4BPα1-2 in cartoon representation. Side chains of amino acids that contact C4BPα1-2 are shown, with labels having the same coloring as in panel a. Dotted lines indicate equivalent amino acids in the M2/M49 and M22/M28 patterns.

Figure S2. Binding of M2 protein constructs to C4BPα1-2.

a. Interaction of $M2_{42}$ and $M2_{53}$ with C4BPα1-2-His at 37 °C, as assessed by a Ni^{2+}-NTA agarose co-precipitation assay and visualized by non-reducing, Coomassie-stained SDS–PAGE. $M22_{248}$ was used as a positive control.

b. Input samples from panel a.

c. Interaction of $GM2_{61}G$ with C4BPα1-2-His, carried out as in panel a.

Each gel in the figure is representative of at least three experimental replicates.

Figure S3. Binding of minimized M2 protein to C4BPα1-2.

Input samples from Ni^{2+}-NTA agarose co-precipitation experiments shown in Figures 1B and C visualized by non-reducing, Coomassie-stained SDS-PAGE (**a,** $M2_{53}$ and $M2_{53}G$; **b,** $M2_{53}G$ and

$M2_{61}G$; $M22_{248}$ was used as a positive control in panels a-b). Each gel is representative of at least three experimental replicates.

Figure S4. Binding of M proteins to C4BP.

Interaction of His-tagged M^{N100} proteins (N-terminal 100 amino acids of mature form) with intact C4BP as assessed by ELISA. C4BP was adhered to the ELISA plate (+C4BP) or not adhered as a negative control (-C4BP), and M proteins were added and detected using an anti-His antibody. All experiments were carried out in triplicate and performed two times. Statistical analysis was performed by one-way ANOVA; $p < 0.05$ *, $p < 0.01$ **, $p < 0.001$ ***, $p < 0.0001$ ****, $p > 0.05$ (not significant, ns).

Figure S5. Titration curves of M2G antiserum against M^{N100} constructs and C4b.

Titration curves fitted to binding of preimmune serum (Pre) or M2G antiserum (Ab) to M^{N100} constructs (N-terminal 100 amino acids of the mature form of the protein) or C4b. **a,** M proteins belonging to the M2/M49 pattern. **b,** M proteins belonging to the M22/M28 pattern. **c,** C4b and M proteins that do not bind C4BP. All data points are shown.

Figure S6. Reactivity of M2G antiserum against human tissues.

Reactivity of M2G and M2 antisera against normal adult human brain tissue lysate (HB) or heart tissue lysate (HH), as determined by western blot analysis. Intact M2 protein (M2) was used as a positive control. Each blot is representative of three experimental replicates. **a,** M2G antiserum (Ab(M2G)). **b,** M2 antiserum (Ab(M2)). **c,** Input samples visualized by Coomassie-stained SDS-PAGE.

Figure S7. Reactivity and crossreactivity of M2G antisera from second rabbit.

Data are presented as in Figure 3.

Figure S8. **ELISA titers of rabbit sera against M proteins.**

Data are presented as in Figure S5.

Figure S9. M2G and M6G competition against M2G antiserum.

a. Binding as determined by ELISA of M2G antiserum to $M2^{N100}$, $M49^{N100}$, $M73^{N100}$, $M28^{N100}$, and $M89^{N100}$, which were adhered to the wells of ELISA plates. Binding was competed with increasing concentrations of M2G or M6G. Experiments were carried out in triplicate three independent times. Means and standard deviations are shown. Statistical analysis for the M2G antiserum was performed by one-way ANOVA; $p < 0.05$ *, $p < 0.01$ **, $p < 0.001$ ***, $p < 0.0001$ ****, $p > 0.05$ (not significant, ns).

b. Binding as determined by ELISA of anti-His antibody to His_6-M6G, which was adhered to wells of ELISA plates. Binding was competed with increasing concentrations of soluble His_6 -M6G. The experiment was carried out in triplicate three independent times. Means and standard deviations are shown, and statistical analysis was as in panel a.

Figure S10. C4BP competition with M2G antiserum.

M2G antiserum was adhered to the wells of ELISA plates, and 0.5 μM His_6-tagged (**a**) $M2^{N100}$, (**b**) $M73^{N100}$, (**c**) $M28^{N100}$, or (**d**) $M89^{N100}$ was added to wells alone (grey), or in the presence of 5 μM fibrinogen (orange) or 0.7 μM C4BP (green). Bound M protein constructs were detected with anti-His antibodies. Experiments were carried out in triplicate three independent times. Means and standard deviations are shown. Statistical analysis for the M2G antiserum was performed by one-way ANOVA; $p < 0.05$ *, $p < 0.01$ **, $p < 0.001$ ***, $p < 0.0001$ ****, $p > 0.05$ (not significant, ns).

Figure S1

a

		abcdefgabcdefgabcdefgabcdefgabcdefg
M2/M49		
**M2**	50	KKEAKLSEAELHDKIKNLEEEKAELFEKLDKVEEE
**M49**	57	VSSVARREKELYDQIADLTDKNGEYLERIGELEER
M73	51	KEAKKLNEAELYNKIQELEEGKAELFDKLEKVEEE
M77	42	EGVSVGSDASLHNRITDLEEEREKLLNKLDKVEEE
M89	49	SVSVKDNEKELHNKIADLEEERGEHLDKIDELKEE
M22/M28		
**M22**	61	ISQESKLINTLTDENEKLREELQQYYALSDAKEEE
**M28**	55	ADKLADAYNTLLTEHEKLRDEYYTLIDAKEEEPRY
M4	52	AWNWPKEYNALLKENEELKVEREKYLSYADDKEKD
M11	55	TNVSADLYNSLWDENKTLREKQEEYITKIQNEETK
M44	50	GSVSLELYDKLSDENDILREKQDEYLTKIDGLDKE
M81	46	ENVPKQQYNALWEENEDLRGRERKYIAKLEKEEIQ

b

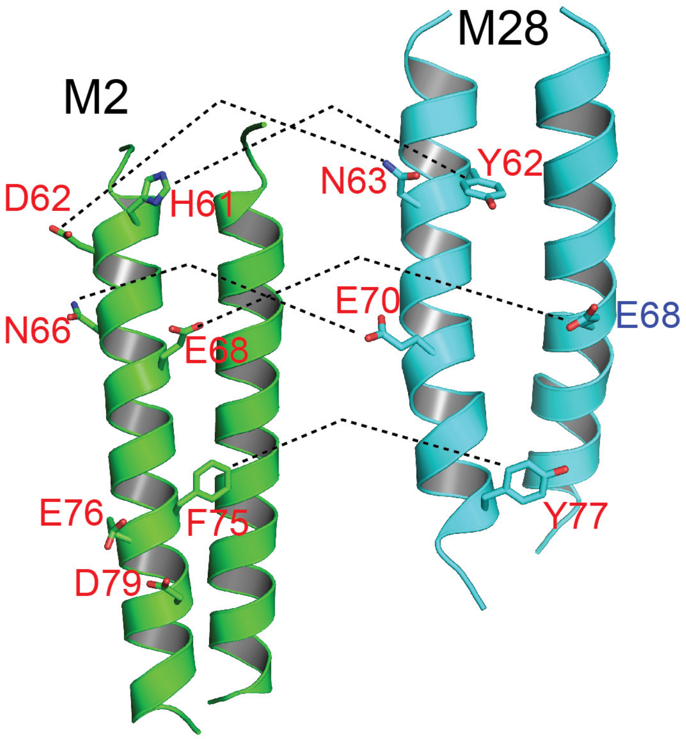

Figure S2

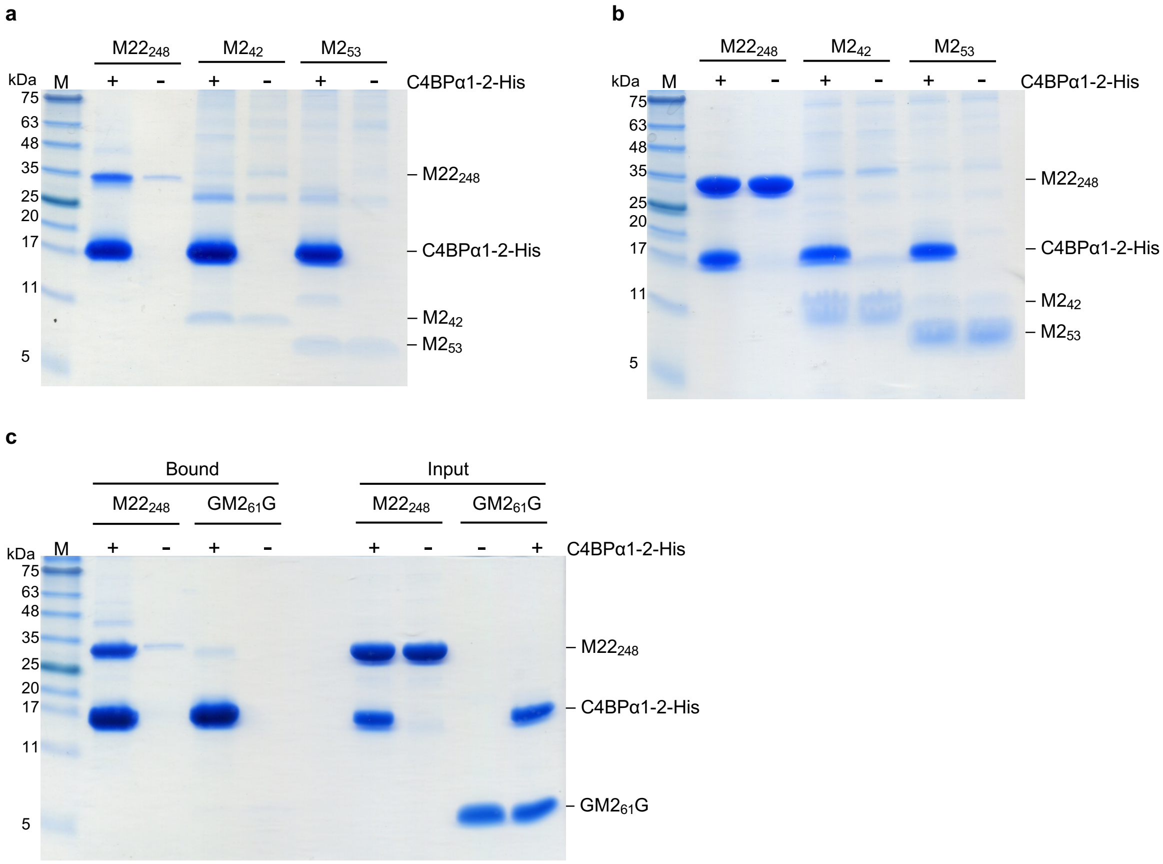

a
M22248
M242
M253
kDa
M
+
-
C4BPα1-2-His
75
63
48
35
25
20
17
11
5
– M22248
– C4BPα1-2-His
– M242
– M253
b
M22248
M242
M253
kDa
M
C4BPα1-2-His
– M22248
– C4BPα1-2-His
– M242
– M253
c
Bound
Input
M22248
GM261G
kDa
M
C4BPα1-2-His
– M22248
– C4BPα1-2-His
– GM261G

Figure S3

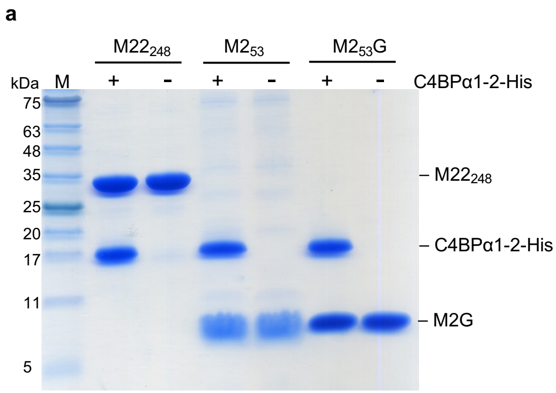
a
M22248
M253
M253G
kDa M
+ - + - + -
C4BPα1-2-His
75
63
48
35
25
20
17
11
5
– M22248
– C4BPα1-2-His
– M2G
b
M22248
M253G
M261G
kDa M
+ - + - + -
C4BPα1-2-His
75
63
48
35
25
20
17
11
5
– M22248
– C4BPα1-2-His
– M2G
– M261G

Figure S4

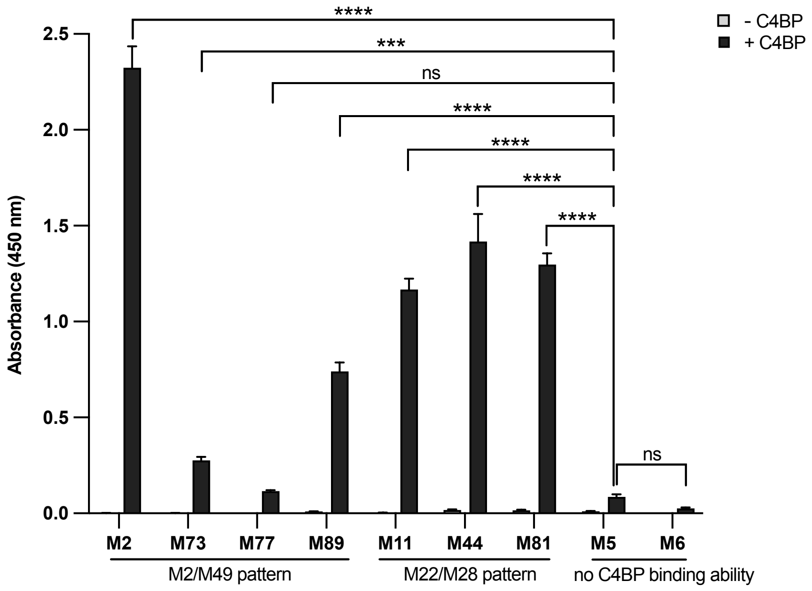

- C4BP
+ C4BP
Absorbance (450 nm)
2.5
2.0
1.5
1.0
0.5
0.0
****
***
ns
****
****
****
****
ns
M2
M73
M77
M89
M11
M44
M81
M5
M6
M2/M49 pattern
M22/M28 pattern
no C4BP binding ability

Figure S5

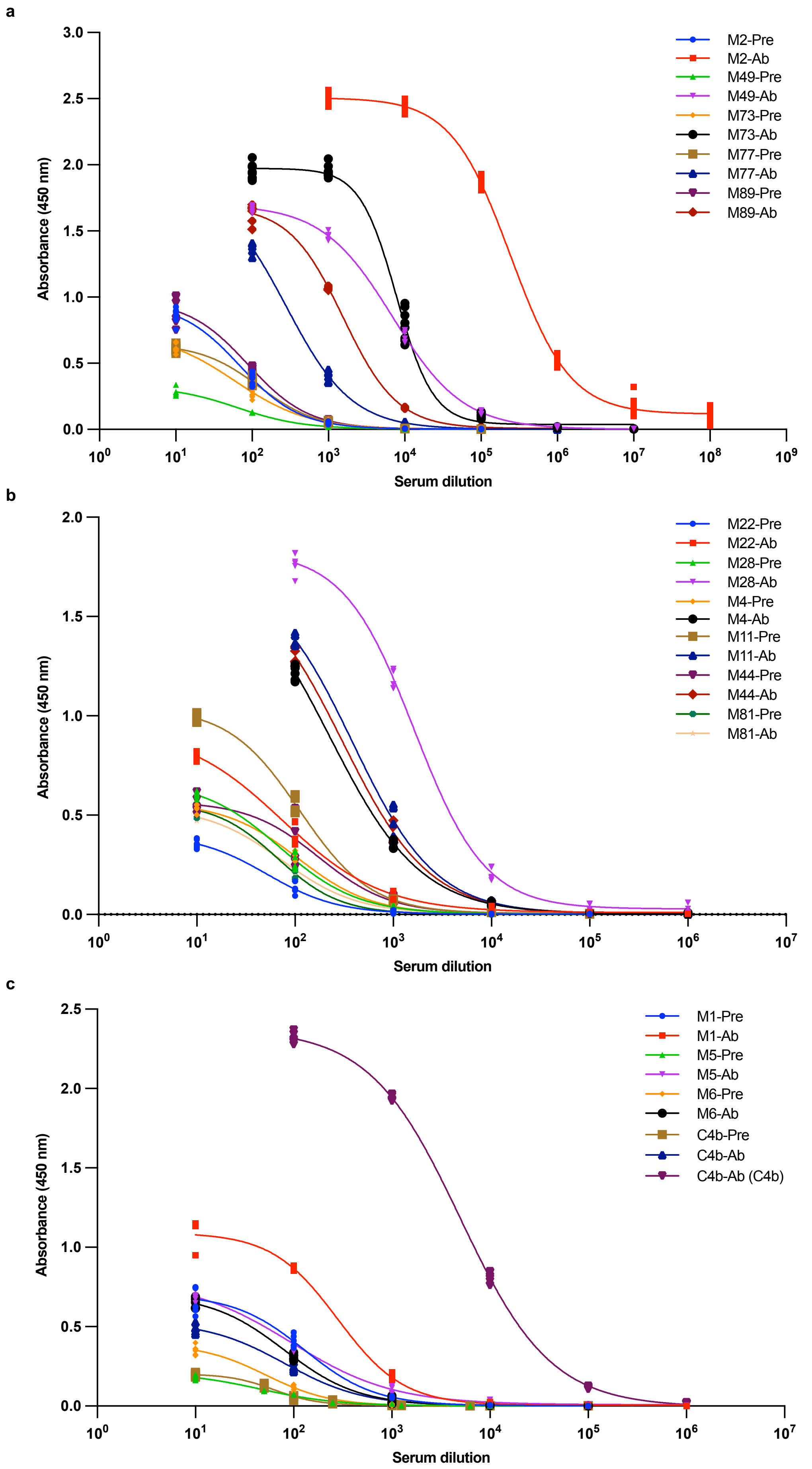
a
Absorbance (450 nm)
Serum dilution
M2-Pre
M2-Ab
M49-Pre
M49-Ab
M73-Pre
M73-Ab
M77-Pre
M77-Ab
M89-Pre
M89-Ab
b
Absorbance (450 nm)
Serum dilution
M22-Pre
M22-Ab
M28-Pre
M28-Ab
M4-Pre
M4-Ab
M11-Pre
M11-Ab
M44-Pre
M44-Ab
M81-Pre
M81-Ab
c
Absorbance (450 nm)
Serum dilution
M1-Pre
M1-Ab
M5-Pre
M5-Ab
M6-Pre
M6-Ab
C4b-Pre
C4b-Ab
C4b-Ab (C4b)

Figure S6

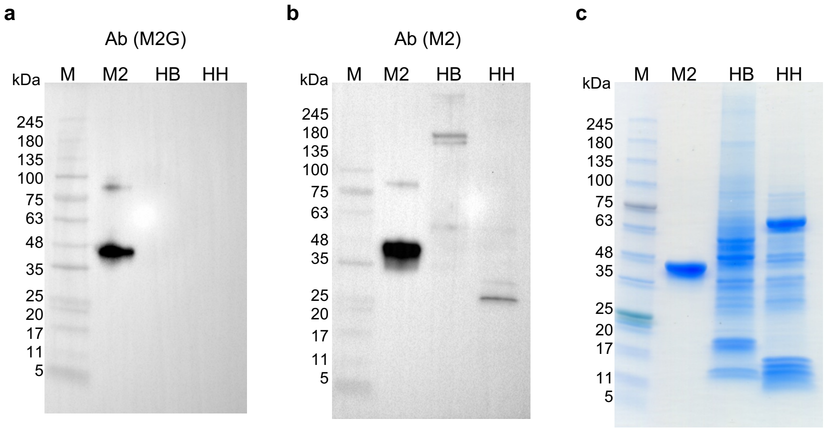
a
Ab (M2G)
kDa
M
M2
HB
HH
245
180
135
100
75
63
48
35
25
20
17
11
5
b
Ab (M2)
kDa
M
M2
HB
HH
245
180
135
100
75
63
48
35
25
20
17
11
5
c
kDa
M
M2
HB
HH
245
180
135
100
75
63
48
35
25
20
17
11
5

Figure S7

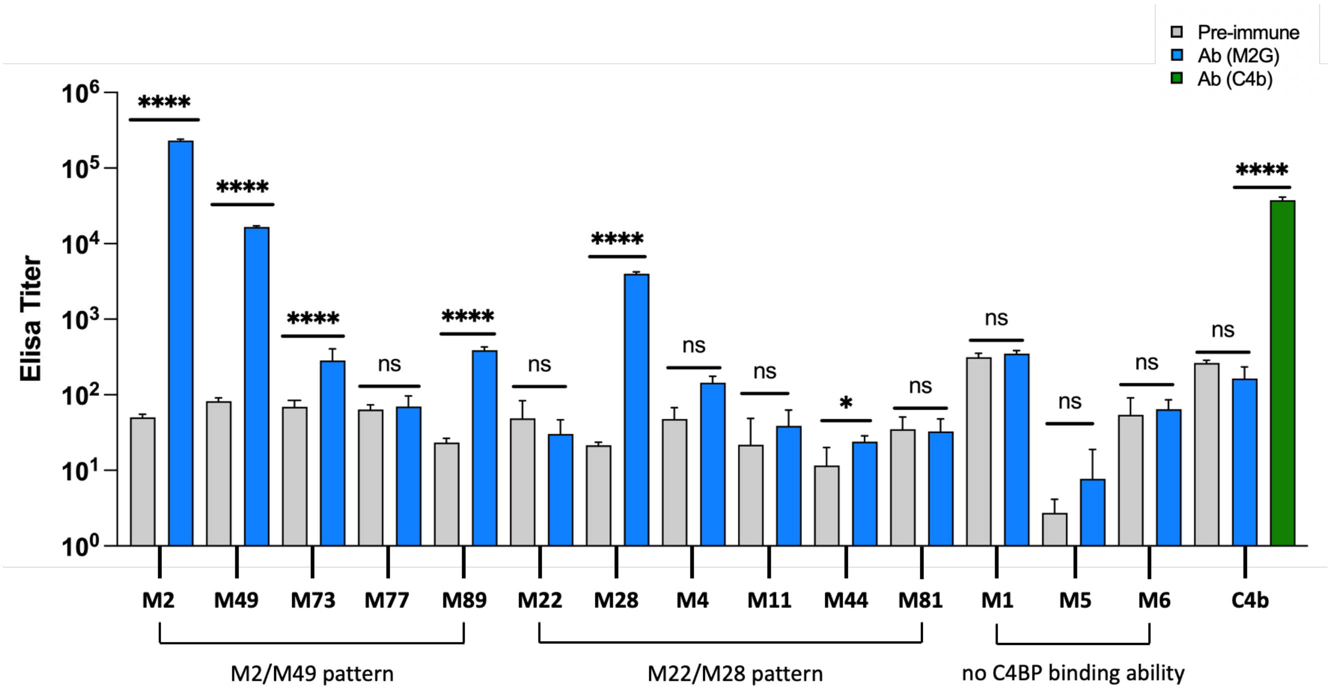
Pre-immune
Ab (M2G)
Ab (C4b)
Elisa Titer
10^6
10^5
10^4
10^3
10^2
10^1
10^0
****
****
****
ns
****
ns
****
ns
ns
*
ns
ns
ns
ns
ns
****
M2
M49
M73
M77
M89
M22
M28
M4
M11
M44
M81
M1
M5
M6
C4b
M2/M49 pattern
M22/M28 pattern
no C4BP binding ability

Figure S8

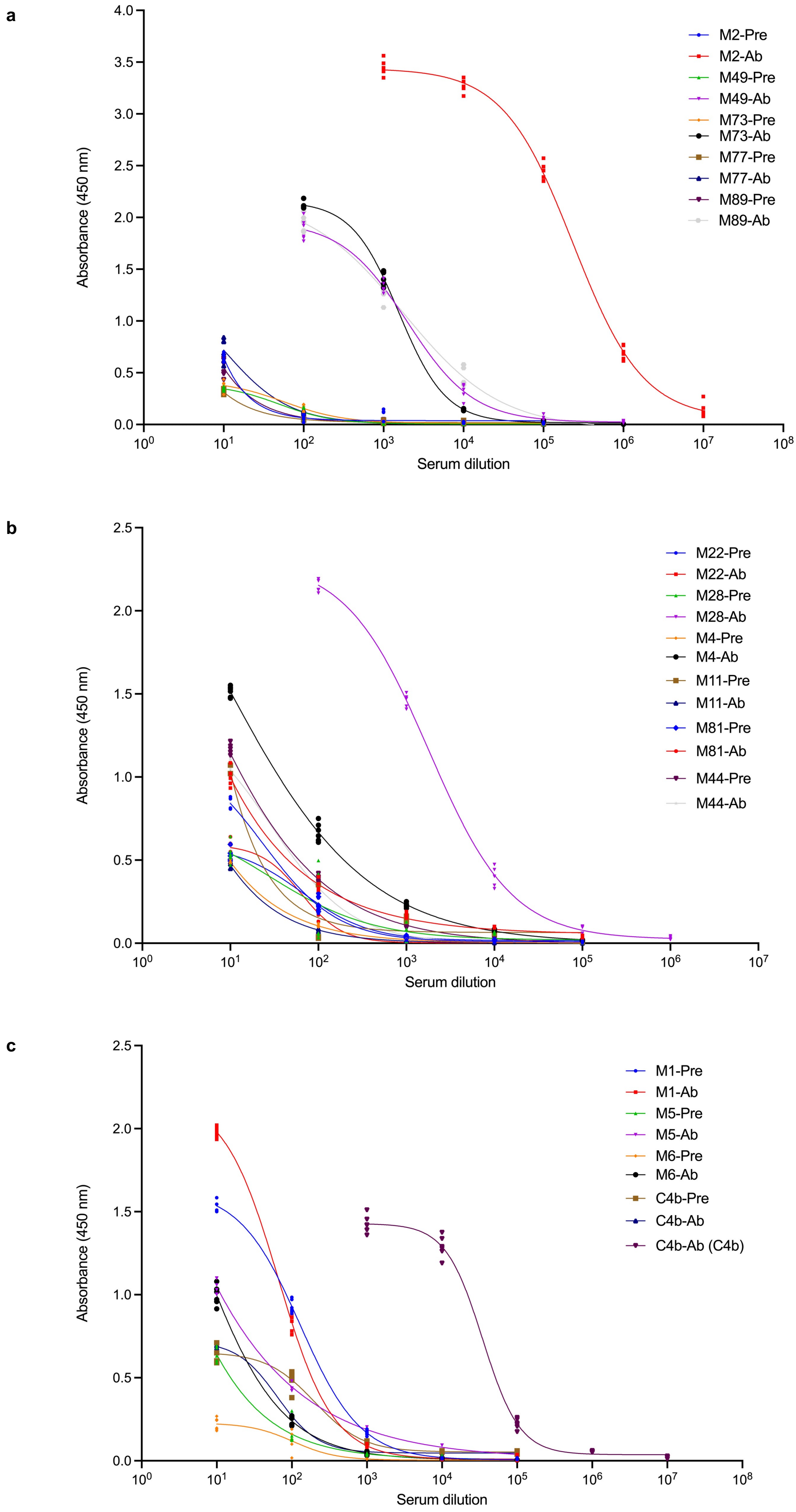
a
Absorbance (450 nm)
Serum dilution
M2-Pre
M2-Ab
M49-Pre
M49-Ab
M73-Pre
M73-Ab
M77-Pre
M77-Ab
M89-Pre
M89-Ab
b
Absorbance (450 nm)
Serum dilution
M22-Pre
M22-Ab
M28-Pre
M28-Ab
M4-Pre
M4-Ab
M11-Pre
M11-Ab
M81-Pre
M81-Ab
M44-Pre
M44-Ab
c
Absorbance (450 nm)
Serum dilution
M1-Pre
M1-Ab
M5-Pre
M5-Ab
M6-Pre
M6-Ab
C4b-Pre
C4b-Ab
C4b-Ab (C4b)

Figure S9

a

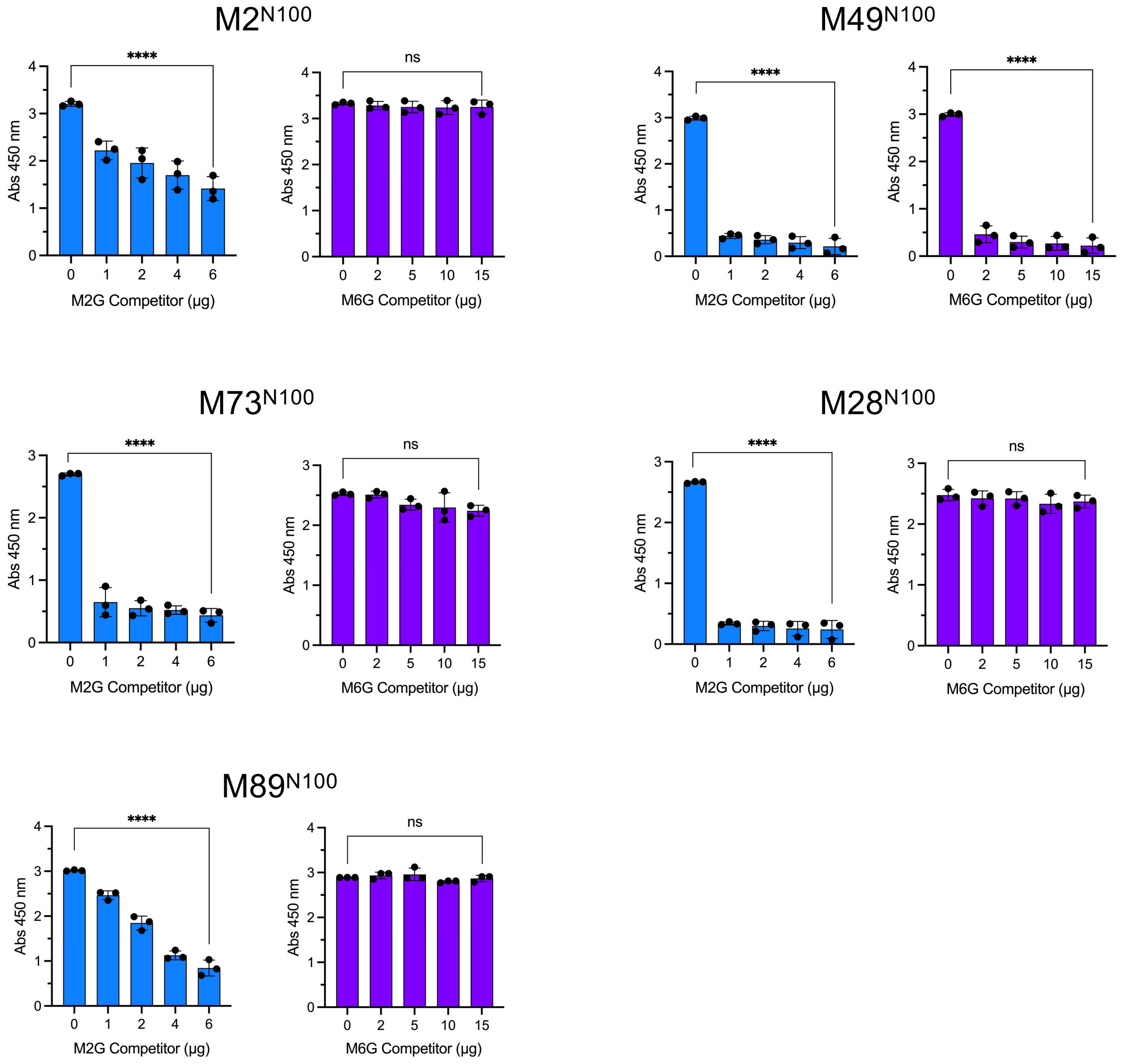
M2N100
M49N100
M73N100
M28N100
M89N100
Abs 450 nm
M2G Competitor (μg)
M6G Competitor (μg)
****
ns
0 1 2 4 6
0 2 5 10 15

b

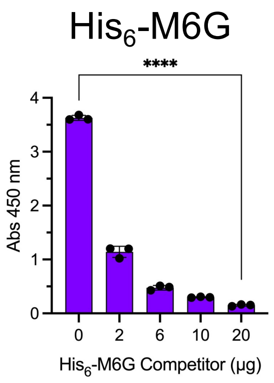
His6-M6G
Abs 450 nm
****
0 2 6 10 20
His6-M6G Competitor (μg)

Figure S10

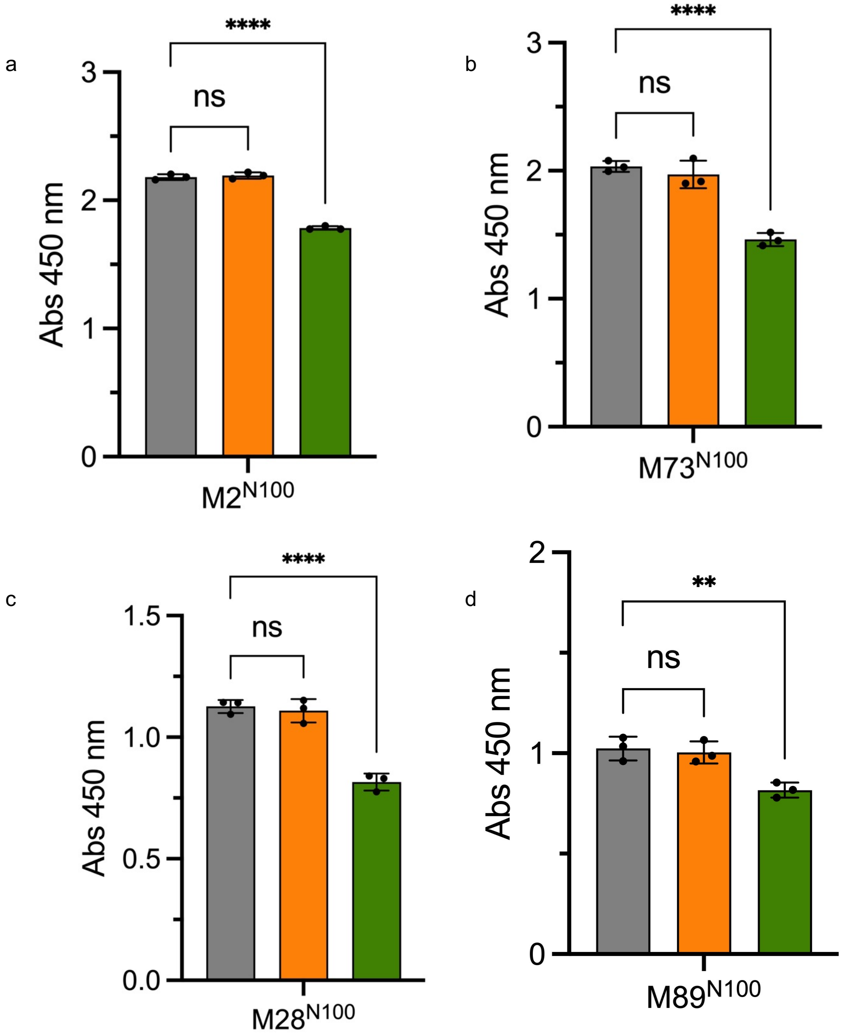
a
Abs 450 nm
0
1
2
3
ns
****
M2^{N100}
b
Abs 450 nm
0
1
2
3
ns
****
M73^{N100}
c
Abs 450 nm
0.0
0.5
1.0
1.5
ns
****
M28^{N100}
d
Abs 450 nm
0
1
2
ns
**
M89^{N100}

Table S1. Protein sequences of M Protein-GCN4 fusion constructs

**GM2$_{61}$G**	E D K V E E L H D K I K N L E E E K A E L F E K L D K V E E E V A R L K K
**M2$_{61}$G**	H D K I K N L E E E K A E L F E K L D K V E E K V E E L L S K N Y H L E N E V A R L K K L V
**M2$_{53}$G**	A K L S E A E L H D K I K N L E E E K A E L F E K L D K V E E E H K Q L E D K V E E L L S K N Y H L E N E V A R L K K L V
**M6G**	A R E L L N K Y D V E N S M L Q A N N D K L T T E N N N L T D Q N K K Q L E D K V E E L L S K N Y H L E N E V A R L K K L V

Amino acid sequences of M proteins and GCN4 portion are marked in red and blue, respectively.

Table S2. Sequence alignment between M2 (aa 53-86) and M proteins (N-terminal 100 amino acids of mature form)

M2 (53-86) vs M protein	Identity (%)	C4BP-binding
M49	32.4	+
M73	70.6	+
M77	58.8	–
M89	50.0	+
M22	35.3	+
M28	35.3	+
M4	29.4	+
M11	23.5	+
M44	32.4	+
M81	26.5	+
M1	26.5	–
M5	23.5	–
M6	29.4	–

Sequence alignment was carried out using LALIGN.
Cross-reactive M proteins are marked in red.

Table S3. Fluorescent intensity of rabbit sera (average of the geometric mean ± standard error)

Strep A strains	Preimmune (pre)	Ab (M2G)	(Ab-pre)/pre
M2	6.1 ± 0.4	194.5 ± 21.8	30.6 ± 1.4
M73	5.7 ± 1.5	25.5 ± 5.8	3.7 ± 0.3
M89	7.6 ± 0.1	12.4 ± 1.1	0.6 ± 0.2
M28	4.8 ± 1.1	13.0 ± 4.4	1.6 ± 0.3
M5	3.6 ± 0.6	4.6 ± 0.8	0.3 ± 0.0
